# The Latent Threat in Wild Birds: *Clostridium botulinum*

**DOI:** 10.3390/vetsci11010036

**Published:** 2024-01-17

**Authors:** Josep Gutiérrez-Arnal, Clara Marín

**Affiliations:** 1 Facultad de Veterinaria, Universidad Cardenal Herrera-CEU, CEU Universities, Calle Santiago Ramón y Cajal 20, 45115 Valencia, Alfara del Patriarca, Spain; josepga21@gmail.com; 2 Departamento de Producción y Sanidad Animal, Salud Pública Veterinaria y Ciencia y Tecnología de los Alimentos, Facultad de Veterinaria, Instituto de Ciencias Biomédicas, Universidad Cardenal Herrera-CEU, CEU Universities, Calle Santiago Ramón y Cajal 20, 45115 Valencia, Alfara del Patriarca, Spain

**Keywords:** avian botulism, *Clostridium botulinum*, wildlife, epidemiological cycle

## Abstract

**Simple Summary:**

This work addresses the significance of *Clostridium botulinum* in avian botulism. Its importance is underscored due to the numerous outbreaks affecting wild birds and the potential risks it poses to biodiversity. To comprehend this pathogen and effectively fight against it, this work has conducted a meticulous compilation of the most cutting-edge scientific studies to date. This allows the reader to gain insights into the most relevant microbiological characteristics of this bacterium, the conditions that most promote its proliferation, its special and effective epidemiological cycle, the avian species most susceptible to it, the most characteristic clinical manifestations and more. All of this is geared towards equipping us with the necessary tools to implement effective surveillance and control strategies, thereby preventing the death of individuals belonging to endangered species through accurate diagnosis and treatment. The aim is to minimise, as far as possible, the presence of this pathogen in the habitats of these birds.

**Abstract:**

Avian botulism caused by *Clostridium botulinum* emerged in 1910, affecting birds across North America, leading to severe outbreaks exacerbated by climate change, decreasing water levels, and inadequate wastewater management. While deadly for birds, its epidemiological impact on humans and other animals remains limited. Despite its significance, understanding and controlling the disease remain challenging. This review delves into the pathogen’s epidemiology in wild bird populations, exploring the transmission, pathogenicity, clinical symptoms, diagnosis and treatment. The disease’s growing concern in wild birds relates to the bacterium’s adaptability and expansive spread, evident through genetic similarities among strains across countries. Outbreaks are influenced by environmental factors such as temperature and soil characteristics. Wild birds inadvertently transmit the bacterium, perpetuating the cycle through carcasses and flies. Some species suffer severely, while others, like scavengers, show resistance. Understanding disease mechanisms, involving potential toxin ingestion or internal production, remains ongoing. Clinical signs vary, affecting diverse bird orders. Diagnostic methods evolve, with treatment success varying among affected populations. Prevention and surveillance take precedence due to treatment challenges, emphasising population-based strategies and preventive measures to manage the widespread presence of *C. botulinum.*

## 1. Introduction

Botulism has become a topic of great relevance due to its significant epizootic potential [1]. Specifically, wild birds represent the primary group of species that suffer from uncontrolled botulism outbreaks due to their numerous concentrations in their habitats and their ability to move to new locations over great distances [2].

In the case of humans and production animals, the disease is fairly well controlled, and in many countries, there is a need for comprehensive monitoring of outbreaks that occur every year [2]. However, in wild animals, specifically in wild birds, the situation is quite different and represents one of the groups with the least control and monitoring of the disease, either due to a lack of direct economic importance or because they do not coexist as closely with humans. This lack of control is what favours the constant and noteworthy outbreaks of avian botulism [3,4]. Understanding the conditions that facilitate its transmission, epidemiological cycle, pathogenicity, susceptible bird species and achieving an accurate diagnosis and treatment are crucial [5]. Developing effective surveillance and control strategies against avian botulism is essential [6]. These strategies could reduce the number of outbreaks in wild birds, lower the mortality rate [6] and prevent possible transmissions to humans and other animals [1].

**Figure 1:**
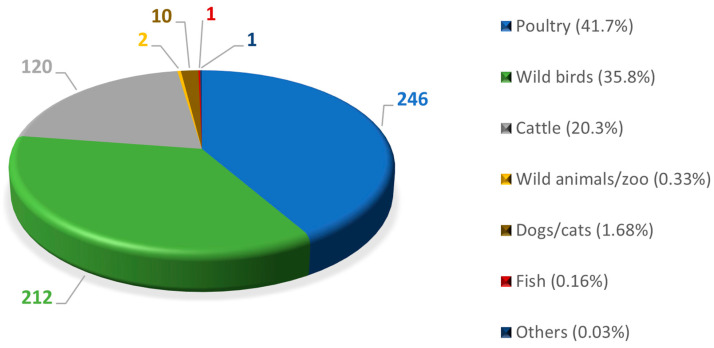
Distribution of botulism cases by species from 2009 to 2019 (592 observed outbreaks). Based on: [2].

Figure 1 represents the significance of botulism in wild birds and other animal species (livestock and pets).

### 1.1. Clostridium botulinum General Characteristics

**Figure 2:**
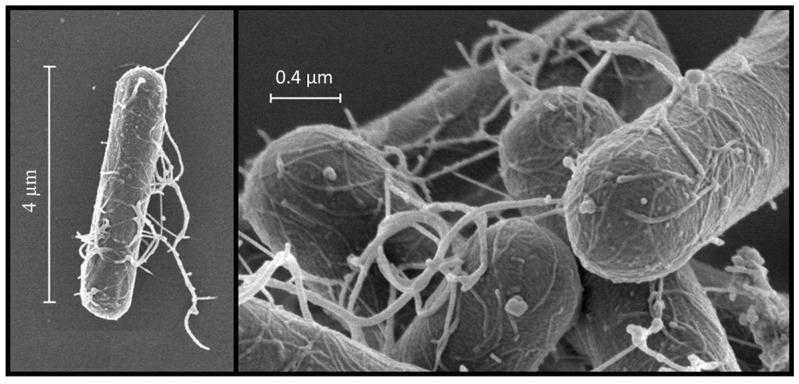
Scanning electron micrograph of *Clostridium botulinum type C/D*, strain BKT015925. Retrieved from: [10].

*Clostridium botulinum* is a species of bacteria from the Clostridiaceae family, which comprises more than 150 species, of which fewer than 20 are pathogenic [7]. These bacteria can be classified according to the mode and site of action of their toxins into several groups, among which the following stand out: histotoxic clostridia, enteropathogenic clostridia and producers of enterotoxaemia, as well as neurotoxic clostridia [8]. This Gram-positive bacillus is characterised by being large in size and having mobility thanks to its peritrichous flagella (Figure 2) [9].

**Figure 3:**
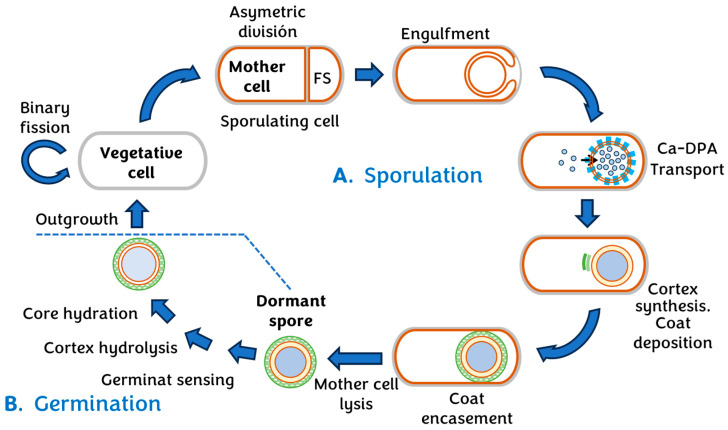
Life cycle of endospore formation in clostridial pathogens. Calcium dipicolinate, Ca-DPA. (**A**) Sporulation. Upon detecting specific environmental conditions endospore formers activate Spo0A and initiate sporulation. (**B**) Germination. Upon detecting the suitable small molecule germinants, the spore initiates a signaling cascade that results in the activation of cortex hydrolases and core hydration. Based on: [13].

Additionally, it is a catalase-negative and oxidase-negative bacterium capable of producing thermoresistant spores and displaying high resistance when exposed to unfavourable conditions for its survival [11]. The serotypes of *C. botulinum* form anaerobic spores that can survive for over 30 years in a liquid medium and probably even longer in a dry environment [12]. These spores require anaerobic conditions and an environment with appropriate nutrients for germination and cell division [11]. This includes high levels of organic material and low oxygen levels, with the optimal growth temperature ranging from 30 to 42 °C [12]. Once the appropriate environmental conditions are met, these spores germinate, giving rise to vegetative cells responsible for the production of BoNT [13]. Figure 3 provides a more detailed view of the complete cycle.

Regarding serotypes, they have been subclassified based on their metabolism and pathogenesis into groups I to IV [1,14]. Groups I (proteolytic strains producing toxins A, B, F) and II (non-proteolytic strains producing toxins B, E, F) include strains pathogenic to humans. Group III strains (producing toxins C or D) are associated with animal botulism and group IV comprises only strains producing neurotoxin G [1,15]. Botulism is typically caused by seven different types of toxins, but, among all of them toxins C and D have been identified exclusively in animals, with D being more prevalent in herbivores and C in birds and carnivores [16].

### 1.2. Hosts and Their Epizootic Potential

Botulism is not a contagious disease, as it is not characterised by direct transmission from a sick animal to a healthy one [1]. Instead, transmission of the disease results from the ingestion of vegetative cells, spores, or BoNT [1]. The lack of direct transmission of *C. botulinum* does not prevent it from being one of the bacteria that presents the greatest diversity of hosts [1,2]. It compensates for this mode of disease dissemination with a high level of resistance and adaptability in the environment, thanks to its spore-forming capability [11]. This ability facilitates its survival over extended periods in the environment [12], thereby increasing the chances of being ingested by a host [12,17]. Furthermore, there are individuals that can act as asymptomatic carriers due to their resistance to certain types of BoNT or to the low number of bacteria in their digestive tract, which is characterised by slow bacterial growth, preventing the production of toxins [5]. These individuals serve as reservoirs for *C. botulinum*, contributing to the spread of the bacteria in their faeces [1,12].

Strains from group III, which include BoNT C and D, give rise to animal botulism [1]. This botulism affects different animal species differently, depending on their sensitivity to BoNT [2]. Birds are more sensitive to BoNT/C and more resistant to BoNT/D [14,18]. It is worth noting that the group of birds, especially wild birds, is the most affected by botulism (BoNT/C) [7]. Almost all wild birds are susceptible to the disease, with very low incidence in vultures, as they are highly resistant [19].

The disease in wild birds has been described over the years on all continents, except Antarctica [4]. However, the first documented outbreaks occurred in the last century in the United States and Canada, affecting populations of wild waterfowl inhabiting wetlands and coastal areas [4]. These outbreaks have not ceased and have continued to be recorded over the past few decades, spreading geographically to Central and South America, Europe, South Africa, Australia and Japan [4]. Such is its current expansion that a significant number of outbreaks have already been recorded in Spain; between 1978 and 2008, the Iberian Peninsula reported 13 outbreaks, resulting in the death of over 20,000 birds and affecting more than 50 species [18]. This had a considerable impact on the populations of wild birds inhabiting the peninsula [18].

## 2. Relevance of Avian Botulism in Wild Birds

Avian botulism has been the cause of numerous outbreaks in wildlife in recent decades, especially in the species that make up the wild fauna [2]. It has wreaked havoc on waterfowl and migratory birds, causing the annual death of thousands, and even millions, of birds, including endangered species, resulting in significant damage to global biodiversity [11,17].

The significance of these outbreaks is related to population density [11,18], as populations with a large number of individuals can withstand losses, whereas species that are endangered or have a restricted geographic distribution are severely affected by a single outbreak, potentially pushing them to the brink of extinction [4]. Some of these birds, such as the Marbled Duck (*Marmaronetta angustirostris*), are classified as endangered on the International Union for Conservation of Nature and Natural Resources (IUCN) Red List, making these outbreaks a significant blow to their populations, since the places where they occur are a crucial nesting site for this duck species [5]. Other notable examples from around the world include the deaths of a significant number of individuals from two endangered species: the Laysan Duck (*Anas laysanensis*) in Hawaii [20] and the Black-faced Spoonbill (*Platalea minor*) in Taiwan [21].

**Figure 4:**
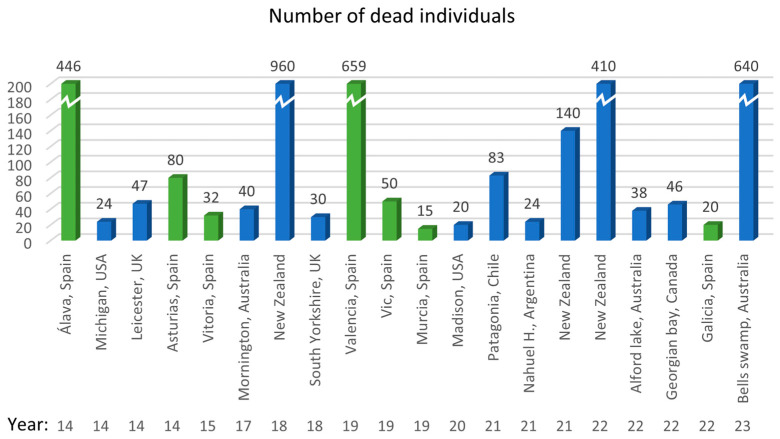
Reported cases of botulism in wild birds distributed by region, country and number of affected individuals. Green represents cases in Spain. Data from: [22,23,24,25,26,27,28,29,30,31,32,33,34,35,36,37,38,39,40].

The record of avian botulism outbreaks worldwide has maintained its incidence in recent years [4], as can be seen in Figure 4, which also indicates the location where it was detected and the mortality it caused in waterfowl.

It is worth noting that, given the spread of this disease in birds, studies have been conducted comparing the strains of *C botulinum type C/D* affecting wild bird populations in Spain with strains from other northern European countries [3]. For instance, there has been a significant genetic similarity observed with Scandinavia, which could be indicative of the remarkable geographical spread potential of this disease [3].

## 3. Situations That Favour *Clostridium botulinum* Transmission

As with other bacteria, there are several environmental factors that facilitate the occurrence of wild bird outbreaks [11,17]. In the case of *C. botulinum*, two of the factors that play a fundamental role in its multiplication are temperature and soil [41]. Regarding temperature, the risk is increased when it rises above 20 °C in wetland water [42], as this bacterium is known to have optimal growth between 25–40 °C [42]. Hence, most outbreaks tend to occur during the warmer months of the year, with the period from August to November (North Hemisphere) being the most likely time for cases [42]. However, outbreaks have also been documented at colder times of the year [17,42], which have been linked to the presence of residual botulinum toxin in the surrounding environment, produced during the preceding warm months [17].

This temperature is also typically accompanied by a basic pH of 7.5–9 in the water and a negative redox potential [11,41]. This is because the temperature increase promotes a multitude of biological processes in the sediment, which, along with the decomposition of organic matter, will ultimately alter the pH and lead to a decrease in the dissolved oxygen levels in the water [11]. Consequently, this will result in a downward modification of the redox potential of the environment [41]. These variations in the environment will benefit the development of *C. botulinum* in the sediment [11,41,43].

Despite these temperature conditions and the presence of poorly oxygenated, shallow waters with organic matter, it is not always an essential requirement for outbreaks of *C. botulinum* to occur [42]. Outbreaks have also been documented in large river systems, deep wetlands and well-oxygenated environments [42].

On the other hand, in numerous natural ecosystems, encompassing agricultural soil, the soil emerges as a pivotal contributor to the proliferation of *C. botulinum* [41]. This bacterium is widely recognised as a common component of the soil microflora [44]. Furthermore, its prevalence exhibits regional variations, with *Clostridium* types C, D and G prevailing prominently in European soils, and notably linked to outbreaks of avian botulism [11]. The prevalence in the substrate is largely attributed to a myriad of physicochemical characteristics, biotic interactions and environmental factors such as temperature and humidity. These factors collectively exert a critical influence on the population dynamics of this pathogen [41]. Moreover, the process of soil-to-plant transfer further accelerates its dissemination [45].

Once the bacteria begin to multiply, to continue the epidemiological cycle, it must spread in order to reach the birds [46]. Birds have several ways of ingesting the toxin [4]. For instance, mallards (*Anas platyrhynchos*) filter sediment to feed, while northern shoveler ducks (*Spatula clypeata*) filter decomposing organic matter from the water [4]. Moreover, numerous invertebrates, such as zooplankton, algae and aquatic plants, have been described that are not affected by BoNT and act as carriers of the toxin and the microorganism [11].

It is worth noting that among all these means of intoxication, the primary culprits in the spread of avian botulism are the maggots of necrophagous flies, through a cycle known as the “Carcass–fly–maggot” cycle [47]. This cycle provides the bacteria with a degree of climatic independence [46,48].

Furthermore, human activities enrich the substrate available for the growth of this bacteria [45]. Drainage in wetlands, the use of pesticides or other contaminants that harm vertebrate and invertebrate animals increase the amount of organic matter, thus creating an optimal substrate for the growth and development of botulinum toxins [17].

## 4. ‘Carcass–Fly–Maggot’ Epidemiological Cycle

Wildlife vertebrates, especially aquatic birds, inadvertently ingest botulinum spores produced by *C. botulinum* while feeding [12]. They act as a means of transport for these resistant forms in their tissues or as asymptomatic carriers that excrete the bacterium in their faeces [42]. Once these vertebrates die, their bodies create an anaerobic environment and a rich source of proteins, which facilitates the germination of botulinum spores, vegetative cell growth and toxin production [49]. Additionally, due to the cadaveric decomposition, there is a temporary increase in temperatures inside the animal, which favours the germination of these spores, thereby granting independence from the external climate, allowing them to germinate in these carcasses even during cold weather seasons [17].

**Figure 5:**
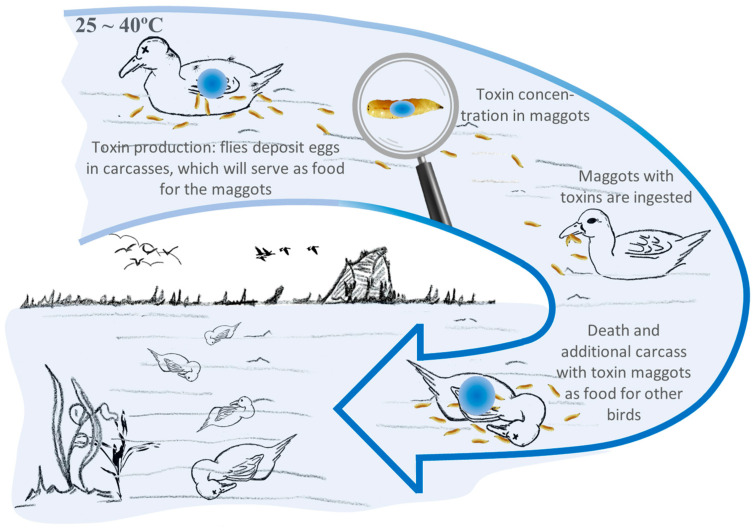
“Carcass–fly–maggot” cycle. Based on: [42].

Cadavers attract and serve as food for necrophagous invertebrates, such as flies, which lay their eggs on them [48]. Later, these eggs hatch into maggots. These maggots are responsible for feeding on decomposing organic matter, accumulating the botulinum toxin without being affected and acting as a transmission vehicle to healthy birds that feed on them [42,47]. In most cases, the maggots disperse beyond the carcasses, thereby increasing the likelihood of being ingested by birds, as many of them do not consume these maggots directly from the carcasses [46,47]. A schematic of the cycle can be seen in Figure 5.

The ingestion of these maggots, depending on the dose, results in clinical outcomes with varying prognoses [4]. Therefore, after ingesting these maggots, birds become ill and die, creating a new suitable substrate for the proper development of *C. botulinum* and its BoNT [42,46]. As the number of deaths increases, the spread of the disease and the number of affected individuals increase exponentially [48], making it a self-perpetuating cycle [11].

This cycle does not stop until environmental conditions are no longer favourable for the growth of bacteria or maggots, which can occur due to a drastic drop in temperatures, human removal of carcasses, predation by other animals, or when birds leave the wetlands [4,50].

It is important to emphasise that this cycle is unique among intoxications because the botulinum toxin generated in the carcasses leads to secondary infection in other individuals, making the epidemiology of avian botulism resemble an infectious disease [7]. Furthermore, *C. botulinum* types C and D have recently been detected not only in maggots and pupae but also in adult flies of the *Calliphoridae* and *Sarcophagidae* families [51]. The presence of the bacterium in adult flies is a new aspect to consider in the epidemiology of avian botulism, as these flies can act as active carriers of *C. botulinum* from one carcass to another [18].

## 5. Wild Birds Most Susceptible to Intoxication

**Figure 6:**
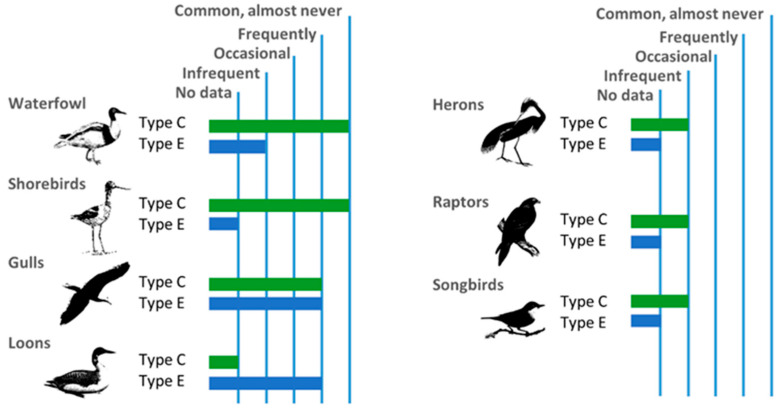
Frequency of botulism cases in the most important groups of wild birds. Based on: [42].

Many populations of wild birds are annually decimated by the effects of *C. botulinum*, with toxins C, D and E being particularly relevant [52]. Figure 6 shows the frequency with which toxins C and E are responsible for outbreaks of avian botulism in different types of birds. Aquatic birds, especially those of the Anseriformes and Charadriiformes orders, are the most affected by the action of this bacterium [5]. They frequently experience numerous outbreaks of avian botulism, often caused by type C [53]. However, in gulls and loons, *C. botulinum* type E takes on a more prominent role [42]. In loons, it is responsible for the majority of outbreaks [43]. In the case of gulls, type E is just as common as type C [42].

On the other hand, we have other wild birds that are less predisposed to population losses due to *C. botulinum* [5]. These include, among many others, diurnal raptors in the Accipitriformes order, nocturnal raptors in the Strigiformes order and other birds in the Passeriformes order, such as songbirds [42]. Furthermore, there are birds with high resistance to toxins produced by *C. botulinum*, such as vultures and other scavenging birds, which have demonstrated resistance to type C [5,54]. This is because they have *Clostridium* and *Fusobacterium* bacteria in their intestinal microbiota that have lost the ability to produce toxins, thus acting as probiotics. They occupy the same niches and consume the same nutrients as the pathogenic strains of *C. botulinum* [19].

## 6. Pathogenicity Factors and the Effects of Their Neurotoxins

The pathogenicity of avian botulism and the factors predisposing wild birds to outbreaks are still under study [55]. However, several hypotheses have been proposed, with the first one suggesting that clinical signs occur due to the direct ingestion of preformed botulinum toxin (BoNT) or ingestion of the bacterium followed by BoNT production in their digestive tract [56]. The second possible scenario, described in poultry, involves the production of BoNT in the caecum. This scenario may be similar in wild birds, as *C. botulinum* has been detected in the digestive tract of diseased individuals [5].

However, once BoNT is ingested or absorbed, due to its structural similarity, it will develop analogous pharmacological action, initiating its neurotoxic effect once it becomes bicatenary molecules [49]. These molecules are directed to cholinergic nerve cells, which will act as the target, exerting a paralysing effect. [57].

It is the catalytic hydrolysis, produced by the action of the light chains of BoNT on the soluble N-ethylmaleimide-sensitive factor attachment proteins (SNARE) in vesicles containing acetylcholine, that blocks the release of the neurotransmitter, resulting in neuromuscular paralysis [58].

## 7. Clinical Manifestations and Diagnosis

Outbreaks of avian botulism are typically characterised by the appearance of rows of carcasses, which coincide in most recorded outbreaks with a drop in the water level [42]. Typically, it is a disease that manifests itself on the water’s edge, and there are rarely sick or dead birds far from the vegetation at the edge [42]. Healthy birds, recently deceased or sick, are usually found grouped in the same areas, with bodies in various stages of decomposition, often affecting species from two, three or even more orders of birds [5].

In general, sick birds remain mentally alert but exhibit symptoms of progressive weakness, paresis and flaccid ascending paralysis of skeletal muscles due to the involvement of peripheral nerves [14,17]. This results in an inability to sustain flight, clumsy walking and in most cases, paralysis of the limbs, with birds often resorting to propelling themselves in water and on land with their wings [14,53].

Furthermore, many individuals have their eyes closed due to paralysis of the nictitating membrane [59]. In the later stages of the disease, the neck muscles become compromised, resulting in an inability to hold the head upright [53]. When birds reach this advanced stage, they often die from drowning or due to cardiorespiratory failure [42]. Among the clinical signs, cases of anorexia or greenish diarrhoea have also been documented, but these are less frequent and nonspecific [53].

The most characteristic and easily recognisable clinical manifestations of avian botulism are the paralysis of the nictitating membrane and the neck muscles [14]. Other signs such as the inability to fly or paralysis of the limbs can be found in other diseases [2], for example, in birds poisoned by lead [42].

On the other hand, the diagnosis is historically based on field signs, clinical symptoms and methods such as those shown in Table 1.

It is worth noting that diagnostic methods are constantly evolving [14]. For instance, the isolation method has successfully isolated 95% of the strains, including 64% of toxigenic strains, when testing type C/D, D, D/C and C samples [14]. Additionally, alternative diagnostic methods based on ELISA and RT-PCR are being explored as substitutes for the mouse bioassay, driven by considerations of animal welfare, cost and time constraints [61].

## 8. Treatment of the Disease

The approach to treatment depends on the type of population, economic availability and the number of affected animals [62]. Treatment of intoxicated birds, especially waterfowl, is generally very successful, with survival rates ranging from 75–90% [42]. However, unlike these, some wild birds, such as coots, gulls or grebes, have a much worse prognosis, even after treatment [42].

Moderately affected birds are treated by providing them with access to water, food and shade [62]. However, in birds with more severe clinical symptoms, an aetiological treatment is considered, which involves the administration of botulinum antitoxin, along with drugs aimed at reducing and preventing more severe symptoms [63]. There have been documented cases of birds in which the injection of type C antitoxin increased the survival rate, even in the presence of both moderate and severe clinical signs [62]. Additionally, when administered in the early stages, a decrease in symptoms is observed [62].

It should be noted that, in most outbreaks, the number of birds affected is very high. Therefore, supportive treatments are often used, which include a combination of vitamin complexes, intravenous fluids, enteral nutrition and antibiotic therapy [5]. Furthermore, the majority of recovered birds remain susceptible to botulinum toxin and, therefore, should be relocated to botulism-free areas to ensure they do not come into contact with the pathogen again [53].

Given all of the above, the control of avian botulism should focus on disease prevention and surveillance rather than treatment [4]. Treatment is costly and challenging to manage when the number of affected birds during an outbreak is very high [62]. However, antitoxin should be available for use in the case of endangered species [63].

## 9. Epidemiological Surveillance and Control Strategies

*C. botulinum* is a ubiquitous bacterium that can be found in both the environment and the digestive tract of animals, making its eradication from the environment practically impossible [7]. Furthermore, due to its complex epidemiology, preventing outbreaks is very challenging [7].

Due to the above, several strategies have been proposed in general to address avian botulism, moving away from individual treatment and focusing on a more population-based approach to reduce losses [6]. One of these measures involves locating areas where outbreaks occur, the time and date of the year and properly recording environmental conditions, which can then lead to implementing a system for surveillance and removal of carcasses [64]. This epidemiological surveillance should begin 10–15 days before the risk dates and end 10–15 days after, thus preventing the “Carcass–fly–maggot” cycle from occurring [42]. At the same time, it is important to prevent abrupt changes in water levels, as this can lead to the death of numerous invertebrates and vertebrates, such as fish, thereby increasing the amount of decomposing organic matter [42]. This increase in organic debris can also be caused by other factors, such as the presence of wastewater, increasing the risk of bacterial proliferation in the environment [7].

According to the Wildlife Health Surveillance Guide of the Hunting Resources Research Institute (IREC), the most effective preventive measure, especially during an avian botulism outbreak, is the collection or incineration of carcasses, with a particular emphasis on vertebrates [65]. To enhance the efficiency of carcass collection, the use of cadaver detection dogs has been implemented, enabling a quicker and more effective detection of water birds in wetlands with dense and flooded vegetation [46].

Additionally, considering the logistical and economic challenges associated with conducting on-site epidemiological studies in many cases, databases of satellite images from areas prone to outbreaks have been developed, supported by artificial intelligence [64]. This approach not only promotes a more precise epidemiological understanding but also provides the opportunity to implement more effective preventive measures [64,65].

## 10. Conclusions

Avian botulism is becoming increasingly relevant in wild birds across different habitats worldwide. Factors such as climate variations, water scarcity, soil characteristics, pollution and the declining population of numerous bird species that contribute to global biodiversity are making recurrent outbreaks of avian botulism an increasingly frequent and latent threat. This has become a significant risk for endangered species.

Therefore, a microbiological understanding of the bacteria, its pathogenicity, situations favouring its proliferation and the most vulnerable groups of wild birds are the fundamental starting points. Alongside a reliable diagnostic method, the establishment of a therapeutic protocol and an effective plan for surveillance and epidemiological control mitigate the number of affected birds and can even prevent outbreaks.

It is essential to emphasize that the most effective approach to address avian botulism primarily focuses on a preventive perspective. This involves implementing control methods and epidemiological surveillance. A more clinical and therapeutic approach is reserved for birds facing extinction or when treatment, based on the number of affected birds, is economically viable. This includes establishing a specific therapeutic protocol with the use of antitoxins.

In recent years, given the growing interest in the conservation of wild birds and significant technological advances, innovative diagnostic techniques that are faster and more reliable have been proposed and developed. These techniques allow for an accurate identification of the causal strain at the population level, leaving behind methods such as the animal bioassay (mouse), which also represents an ethical advancement. One of the technological breakthroughs that could mark a turning point in disease surveillance and epidemiological control is the use of artificial intelligence. It serves as a preventive method that, with proper implementation, can significantly reduce the number of outbreaks and affected wild birds, enabling proactive planning and cost reduction.

## Figures and Tables

**Table 1:** Comparative Diagnostic Methods for *Clostridium botulinum*. Compiled with data from: [3,60,61].

Diagnosis Method	Description	Advantages (A) or Disadvantages (D)
Mouse bioassay	Intraperitoneal inoculation with a suspicious sample in mouse 1, and neutralisation with polyvalent antitoxin or boiling in mouse 2. If (+), mouse 1 shows clinical signs (ruffled fur, posterior third paralysis, wasp waist, death, etc.). Mouse 2 shows no signs. If (−), both mice survive. Types the BoNT of the outbreak if a neutralisation test is done with specific antitoxin.	(A): Detection of toxin in serum, biological samples (stool, food and gastric contents), environmental samples (sediments) and crops. (A): High sensitivity and specificity. (D): Detection takes one to several days. (D): Ethical issues. (D): Difficult interpretation of results due to nonspecific signs in mice or prior death.
Cultivation and isolation	1º. Cultivation in liquid medium “chopped-meat-glucose-starch”. 2º. PCR confirmation of *Clostridium Botulinum.* 3º. If (+), solid medium culture, “blood agar”.	(D): Non-selective culture media. (D): Some strains lose the “bot” gene phage that encodes toxins, preventing their characterisation in mouse bioassays or PCR.
ELISA	It is carried out using polyclonal antibodies against a semi-purified toxic complex of BoNT.	(A): Analyses greater nº of samples than bioassay. (D): Less sensitive and specific than the bioassay. (D): False + due to the detection of inactive toxins that cross-react with other toxins. (D): False − due to the genetic variability of toxins.
Real-time PCR	1º. Sample enrichment in a culture broth to germinate the spores, increase the number of microorganisms and dilute inhibitors. 2º. Performance of PCR where millions of copies of specific target genes of the microorganism are produced.	(A): Sensitive and specific. Faster than culture and bioassay; allows analysis of more samples. (A): Enables ecological and epidemiological studies. (A): Detects BoNT C, D in environment and tissues. (D): Only reverse transcriptase PCR detects gene and toxin activity. (D): False + due to the detection of dead cells. (D): False − due to loss of the “bot” gene.
Mass spectrometry	In vitro detection of the peptides that form botulinum toxins after cleaving SNARE proteins at specific points.	(A): Detects biologically active toxin. (A): High sensitivity and specificity. (D): Only commercially available for type A toxins. (D): Expensive equipment & specialised personnel.

## Data Availability

Data are contained within the article.
